# Fretting Corrosion Behavior of Experimental Ti-20Cr Compared to Titanium

**DOI:** 10.3390/ma10020194

**Published:** 2017-02-17

**Authors:** Tomofumi Sawada, Christine Schille, Atif Almadani, Jürgen Geis-Gerstorfer

**Affiliations:** 1 Section Medical Materials Science & Technology, University Hospital Tübingen, Osiander Strasse 2-8, Tübingen 72076, Germany; christine.schille@med.uni-tuebingen.de (C.S.); Atif.Almadani@med.uni-tuebingen.de (A.A.); geis-gerstorfer@mwt-tuebingen.de (J.G.-G.); 2 Department of Prosthodontics, Center of Dentistry, Oral Medicine, and Maxillofacial Surgery, University Hospital Tübingen, Osianderstrasse 2-8, Tübingen 72076, Germany

**Keywords:** titanium, titanium alloys containing chromium, Ti-20Cr, fretting corrosion test, passive oxide film, repassivation time, scratching

## Abstract

Experimental cast titanium alloys containing 20 mass% chromium (Ti-20Cr) show preferable mechanical properties and a good corrosion resistance. This study evaluated the fretting corrosion behavior of Ti-20Cr. Ti-20Cr (*n* = 4) and commercially pure titanium (CP-Ti, *n* = 6) disk specimens were used. The fretting corrosion test was performed by electrochemical corrosion at 0.3 V in 0.9% saline solution and mechanical damage using 10 scratching cycles with three different scratching speeds (10–40 mm/s) at 10 N. After testing, the activation peak, repassivation time and surface morphology of each specimen were analyzed. The differences between the results were tested by parametric tests (α = 0.05). The average activation peaks were significantly higher in CP-Ti than in Ti-20Cr (*p* < 0.01), except at 20 mm/s. In the series of scratching speeds, faster scratching speeds showed higher activation peaks. The maximum activation peaks were also higher in CP-Ti. Slight differences in the repassivation time were observed between the materials at every scratching speed; faster scratching speeds showed shorter repassivation times in both materials (*p* < 0.05). CP-Ti showed severe damage and significantly higher wear depth than Ti-20Cr (*p* < 0.05). In conclusion, adding chromium to titanium reduced surface damage and improved the fretting corrosion resistance.

## 1. Introduction

Titanium has been used in dentistry for more than half a century [1]. Titanium and its alloys are widely used as biomaterials due to their superior mechanical properties [2,3,4,5]. The advantages of these materials include excellent biocompatibility, corrosion resistance, and low metal hypersensitivity compared with other metals [3,4]. Thus, commercially pure titanium (CP-Ti) and Ti-6Al-4V are primarily used in dental implant treatments [2]. The application of these materials will increase in the future because of the increasing patients’ demands for dental implants to reconstruct their oral function due to missing teeth [5,6,7].

However, these materials cause some clinical problems under certain conditions. For instance, discoloration and dissolution of CP-Ti and Ti-6Al-4V were observed in denture frameworks and orthodontic brackets due to inappropriate usage of strong alkaline denture cleansers and fluoride-containing prophylactic products, respectively [3,4,8]. Generally, high corrosion resistance in these materials is mainly achieved by a thin metal oxide passive film [4,9]. The primary cause of these corrosion behaviors is destruction of the passive film in the presence of fluorides or peroxides under acidic conditions [10]. Furthermore, dissolution of Al and V elements in Ti-6Al-4V results in the potential risk of toxicity for the human body [4]. Therefore, patients who use these titanium products should pay attention to such detrimental effects.

For achieving good corrosion resistance in such cases, experimental cast titanium alloys containing 10 mass% copper (Ti-10Cu) or 20 mass% chromium (Ti-20Cr) have been attempted [11,12]. Although Ti-10Cu had antibacterial effects against *S. aureus* and *E. coli*, the corrosion reaction could unfortunately be promoted under acidic conditions and in the presence of fluorides [11,13]. By contrast, titanium-chromium alloys (Ti-Cr) had greater resistance to electrochemical corrosion in fluoride-containing artificial saliva compared to CP-Ti and Ti-6Al-4V [14]. In addition, Ti-20Cr reduced discoloration and dissolution by immersion in peroxide- or fluoride-containing solutions compared to CP-Ti and other titanium-based alloys [10]. It has already been clarified that formation of a chromium oxide–rich surface film improves corrosion resistance to fluoride in Ti-Cr [15]. From these laboratory findings, for the biological aspects, Ti-20Cr may be expected to find application as a preferred material for dental fixed prostheses and dental implant abutments.

Titanium is corrosion-resistant under controlled environments in the absence of load [16]. In addition, dissolution of titanium-based alloys is a rare phenomenon under the ideal conditions of passivity with no damage to the dental implant surface according to a previous review [17]. However, the authors mentioned that corrosion of these implants may occur when the oral conditions are unfavorable such as under mechanical trauma. Other researchers have then attempted to investigate not only the chemical corrosion of titanium but also the effect of simultaneous chemical and mechanical action (tribocorrosion behavior) on the corrosion properties, as expected in the human body’s internal environment [18,19,20], because the metal oxide passive film is abraded due to physical surface damage such as mechanical fretting action [9]. Moreover, the synergistic effect of electrochemical corrosion damage and mechanical scratching damage is dependent on the scratching speed [20]. In view of this degradation process, it is still unclear how mechanical action influences the corrosion behavior of Ti-20Cr.

The aim of this study was to test the fretting corrosion behavior of Ti-20Cr by using a chewing simulator with different scratching speeds in 0.9% saline solution. The null hypothesis was that Ti-20Cr would not improve corrosion resistance compared with CP-Ti in the series of scratching speeds. However, the experimental results indicated that the addition of chromium to titanium resulted in less surface damage and improved the fretting corrosion resistance.

## 2. Materials and Methods

### 2.1. Preparation of Specimens

For this study, disk samples (diameter; 10 mm, thickness; 1.5–2.0 mm, and surface area; 0.79 cm^2^) were prepared from cast CP-Ti (grade 2, *n* = 6) and Ti-20Cr (*n* = 4). Ti-20Cr ingots, which were obtained from the Department of Dental Materials Science, Tokyo Dental College (Tokyo, Japan), were made from the melting sponge titanium (Ti; >99.8 mass%, OSAKA Titanium technologies Co., Ltd., Amagasaki, Japan) and pure chromium (Cr; >99.99 mass%, Japan Metals & Chemicals Co., Ltd., Tokyo, Japan) [15]. In fact, this cast alloy was turned over six times during melting to make homogeneous ingots [21]. The respective ingots were arc-melted and cast into the mold using an argon-arc melting/pressure cast machine to prepare the samples of both CP-Ti and Ti-20Cr by Dentaurum GmbH (Ispringen, Germany) according to previous literatures [15,21]. The rear side of each sample was connected with a titanium wire (rematitan 0.5, Dentaurum GmbH, Ispringen, Germany) by laser welding for electrical contact. Each wire was then covered with a shrinkable tube for electrical isolation. Each sample was embedded in an acrylic resin block (Palavit G, Heraeus Kulzer GmbH, Wehrheim, Germany). Prior to the fretting corrosion test, all specimens were grinded by carbide papers (CarbiMet, Buehler-Wirtz, Düsseldorf, Germany) until #1200 grit and ultrasonically cleaned with ethanol for 5 min.

### 2.2. Fretting Corrosion Test

**Figure 1:**
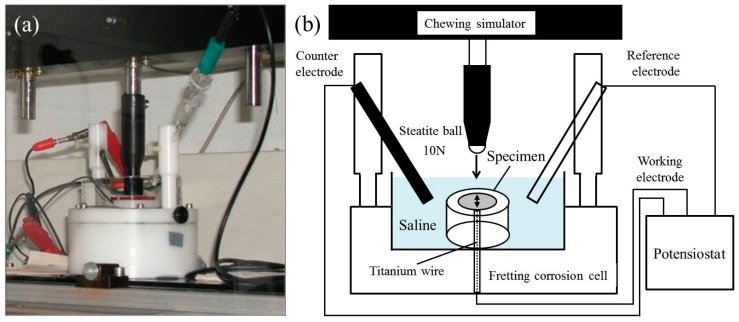
(**a**) Fretting corrosion cell; (**b**) A schematic diagram of the fretting corrosion test.

**Figure 2:**
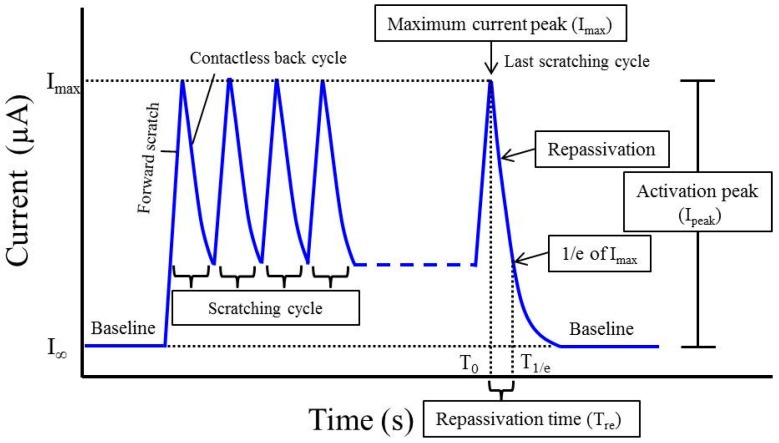
A typical fretting corrosion plot.

A special fretting corrosion cell, which was made from polyacetal (POM), was fixed in a chewing simulator (Willytec, SD Mechatronik GmbH, Feldkirchen-Westerham, Germany), and each specimen was then placed onto the cell (Figure 1a). Electrochemical corrosion was performed using a potentiostat (Gamry PC4, Software: LV500, C3 Prozess- und Analysentechnik GmbH, Haar, Germany) with a three-electrode assembly combined with two sense electrodes [20]. Each specimen was used as the working electrode and the counter and reference electrodes were a graphite rod and silver/silver chloride (Ag/AgCl), respectively (Figure 1b). The cell was filled with 0.9% saline solution as the electrolyte and each specimen was immersed for 10 min to obtain the equilibrium electrode potential for each specimen. A constant potential was applied at 0.3 V (REF) over 10 min [12,22]. After recording the baseline for 5 min, 10 scratching cycles on each specimen were performed with the chewing simulator using a ball-on-disk wear method. A steatite ball (diameter; 6 mm) was used as an antagonist at 10 N [23,24]. The scratching damage was set at a 2 mm length by a linear sliding frictional wear. As a result of the oxide removal, an increase in the corrosion current occurs. A typical fretting corrosion plot is represented in Figure 2. At the end of the scratch path the antagonist was lifted and returned to the starting position. During this contactless back cycle, titanium oxide is again formed (repassivation), which led to a current reduction. In this study, three different scratching speeds (10, 20, and 40 mm/s) were used for both CP-Ti and Ti-20Cr specimens at room temperature.

In all fretting corrosion measurements, each maximum current peak and the baselines before and after scratching test were recorded. Each activation peak (I_peak_) was calculated from the difference between the baseline (I_∞_) and the maximum current peak (I_max_) by intensity of current in units of μA using the following Equation (1) (Figure 2):

I_peak_ = I_max_ − I_∞_ (1)

Usually, it is assumed that the passivation is completed when the baseline is reached again. Since this cannot always be reliably determined, the repassivation time (T_re_) for each specimen was calculated according to a previous literature [25]. In brief, the repassivation time was defined as the difference between the times of the maximum current peak (T_0_) and 1/e of the maximum current peak (T_1/e_) in the last scratching cycle using the following Equation (2) (Figure 2):

T_re_ = T_1/e_ − T_0_ (2)

### 2.3. Surface Morphology

After fretting corrosion test, the scratching depth in each specimen surface was measured by a profilometer (Perthometer SP6, Mahr GmbH, Göttingen, Germany) using a topographic plot with 101 profiles which was set to 3 × 3 mm. The profile depth (Pt) in the specimens was calculated. The surface morphology in the scratched area of each specimen was then observed by stereomicroscopy (M400 Photomicroscope, Wild Heerbrugg AG, Heerbrugg, Switzerland).

### 2.4. Statistical Analysis

All data were analyzed for normal distributions by Shapiro-Wilk test and for variance equality by Levene test. The results of repassivation time, average of activation peaks, and scratching depth were then analyzed by two-way analysis of variance (ANOVA) with scratching speed and scratching phase or metal alloys as independent factors, followed by Tukey’s test for post-hoc comparisons. Statistical analyses were performed by using the software package Excel Statistics 2010 (Social Survey Research Information Co., Ltd., Tokyo, Japan) at a level of significance of α = 0.05.

## 3. Results

### 3.1. Current Intensity

**Figure 3:**
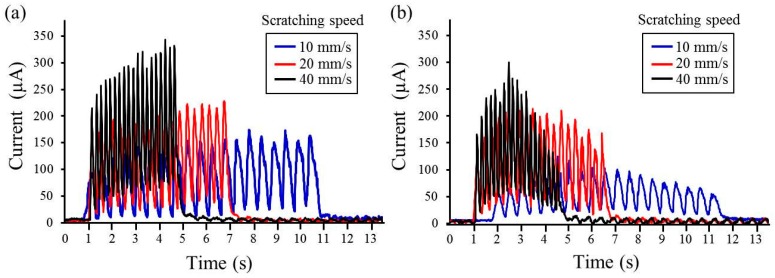
Fretting corrosion plots for both commercially pure titanium (CP-Ti) and titanium alloys containing 20 mass% chromium (Ti-20Cr) at different scratching speeds: (**a**) CP-Ti; (**b**) Ti-20Cr.

**Figure 4:**
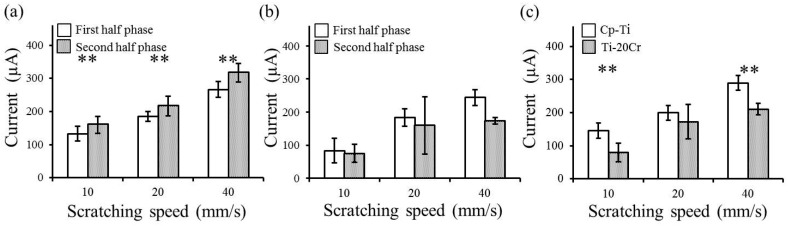
Average values of activation peaks in commercially pure titanium (CP-Ti) and titanium alloys containing 20 mass% chromium (Ti-20Cr) at different scratching speeds: (**a**) CP-Ti, (**b**) Ti-20Cr (**c**) Total phase (first + second half-phase). First and second half-phases indicate one to five and six to 10 scratching cycles, respectively. The asterisk indicates statistical significance of the difference between groups (*p* < 0.01).

Fretting corrosion plots for both CP-Ti and Ti-20Cr, corresponding to different scratching speeds, are shown in Figure 3. The maximum activation peaks in CP-Ti were higher than those in Ti-20Cr at every scratching speed. Faster scratching speeds showed higher peak values in both materials; however, their increasing behaviors were different. Average values of the activation peaks for the first half-phase (from one to five scratching cycles) and second half-phase (from six to 10 scratching cycles) of scratching damage application are shown in Figure 4. In the two-way ANOVA results of the average of the activation peaks, the interactions between the scratching speed and scratching phase (Figure 4a,b) or alloy type (Figure 4c) were not statistically significant (*p* = 0.4772, 0.4445 and 0.1504, respectively). In CP-Ti, the activation peaks gradually increased and finally reached the maximum values (Figure 3a). Therefore, the activation peaks in the first half-phase were significantly lower than those in the second half-phase at the series of scratching speeds in CP-Ti (*p* < 0.01, Figure 4a). In contrast, the activation peaks in Ti-20Cr achieved the maximum values at the intermediate point and then decreased (Figure 3b). There were no significant differences in the activation peak values between the first and second half-phases in Ti-20Cr (Figure 4b). In the total phase, the average value of the activation peaks also increased with the increase of the scratching speed in both materials (Figure 4c). There were significant differences in the average value of the activation peaks at every scratching speed between CP-Ti and Ti-20Cr (*p* < 0.01), except at 20 mm/s.

**Figure 5:**
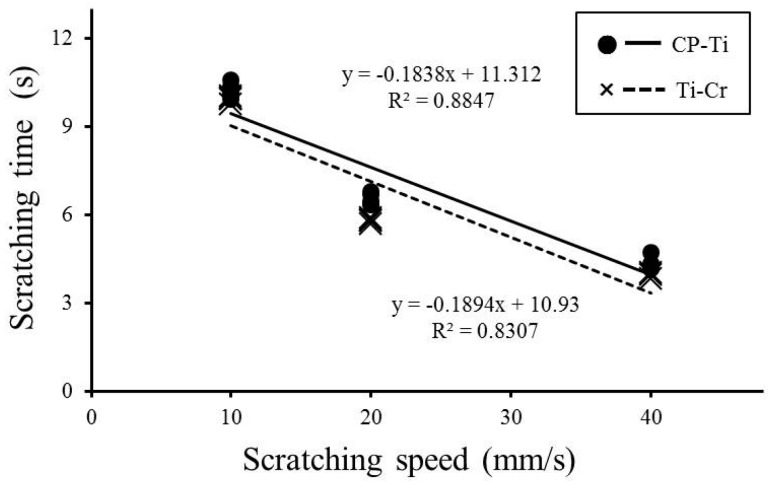
Linear regression between the scratching time and scratching speed for both commercially pure titanium (CP-Ti) and titanium alloys containing 20 mass% chromium (Ti-20Cr).

The correlations between the scratching time and scratching speed for both CP-Ti and Ti-20Cr are shown in Figure 5. The scratching times during the fretting corrosion test between the baselines before and after the scratching damage in CP-Ti and Ti-20Cr were 10.22 and 9.93 s, 6.52 and 5.81 s, and 4.33 and 3.95 s at scratching speeds of 10, 20 and 40 mm/s, respectively (Figure 5).

### 3.2. Repassivation Time

Repassivation times calculated from the last scratching cycle for both CP-Ti and Ti-20Cr at different scratching speeds are shown in Table 1. The interactions between the alloy type and scratching speed were observed in the two-way ANOVA result of the repassivation time (*p* = 0.0021). From the main effects analysis, the repassivation times for CP-Ti were significantly shorter than those for Ti-20Cr at the 10 mm/s scratching speed (*p* < 0.05) whereas the differences at other scratching speeds were not significant. Faster scratching speeds showed shorter repassivation times in both materials (*p* < 0.05).

### 3.3. Surface Morphology

**Figure 6:**
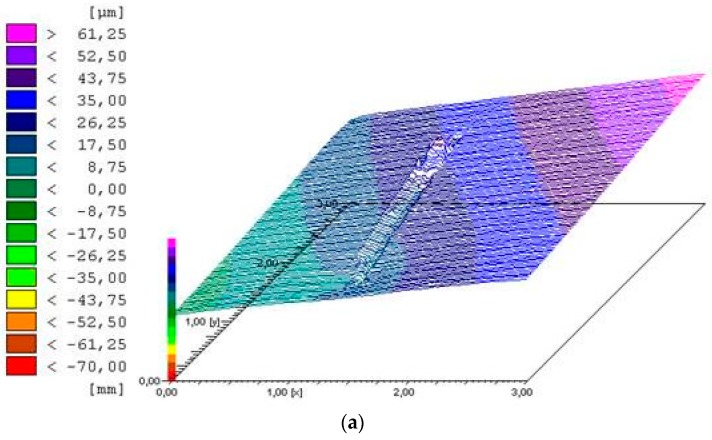

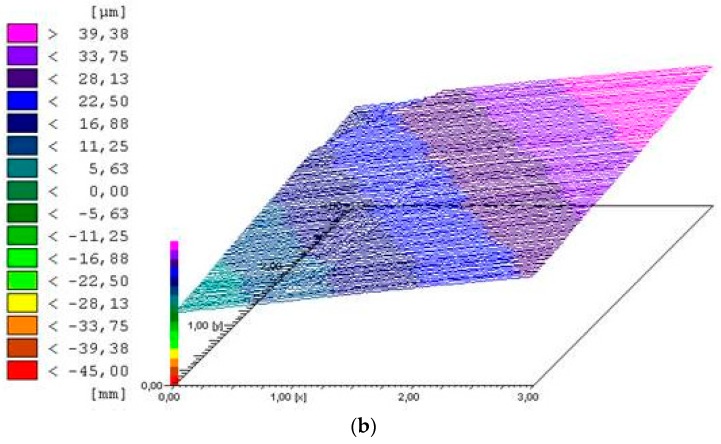
Surface topography of both commercially pure titanium (CP-Ti) and titanium alloys containing 20 mass% chromium (Ti-20Cr) at 10 mm/s scratching speed: (**a**) CP-Ti; (**b**) Ti-20Cr.

**Figure 7:**
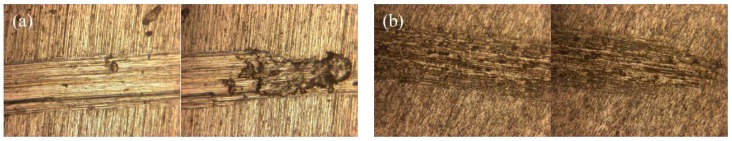
Microscope image (100× magnification) of the scratched areas in both commercially pure titanium (CP-Ti) and titanium alloys containing 20 mass% chromium (Ti-20Cr) at 10 mm/s scratching speed: (**a**) CP-Ti; (**b**) Ti-20Cr.

The results for Pt are shown in Table 2. The interactions between the alloy type and scratching speed were observed in the two-way ANOVA result of the scratching depth (*p* = 0.0076). From the main effects analysis, the average values of Pt in CP-Ti were significantly higher than those in Ti-20Cr (*p* < 0.05), irrespective of the scratching speed. These differences were confirmed by topography and microscopy observations, as shown in Figure 6 and Figure 7, respectively. The surface damage was clearly confirmed in CP-Ti (Figure 6a and Figure 7a) while only slight wear damage by fretting corrosion was observed in Ti-20Cr (Figure 6b and Figure 7b).

## 4. Discussion

This study focused on the fretting corrosion behavior of Ti-20Cr compared to that of CP-Ti. Ti-20Cr showed lower maximum activation peaks compared to CP-Ti, irrespective of the scratching speed. The average activation peaks in Ti-20Cr were significantly lower compared to those in CP-Ti (*p* < 0.01), except at the 20 mm/s scratching speed. Remarkable differences in the repassivation times between CP-Ti and Ti-20Cr were not observed. The activation peaks showed different kinetic behaviors. These differences were also confirmed from the topographic analysis, resulting in the deeper wear damage in CP-Ti and slight damage in Ti-20Cr. These findings imply that Ti-20Cr showed less surface damage and improved the fretting corrosion resistance compared to CP-Ti. Thus, our null hypothesis was rejected.

The corrosion behaviors and mechanical properties of Ti-Cr are influenced by the chromium content [15,21]. Generally, titanium and its alloys show corrosion resistance due to a passive surface oxide film [26]. In particular, alloys with chromium must contain a minimum of 12 mass% of this element to achieve an adequate passive film [27]. The surface layer of Ti-Cr consists of different compositions of titanium and chromium oxide films depending on the chromium content. According to a previous study [15], increasing the chromium content from 5 to 20 mass% significantly prevented the dissolution of both titanium and chromium against NaF solution in Ti-Cr compared to that in CP-Ti. Takemoto et al. [15] clarified the fact that fluoride attack mainly caused selective corrosion in titanium oxide film in Ti-Cr because chromium oxide has good resistance to fluoride-induced corrosion. Moreover, the addition of 15 or 20 mass% chromium to titanium, which is in the range of hypereutectoid composition, resulted in sufficient elongation values and high yield- and tensile- strengths because the eutectoid composition is approximately 13.5 mass% [21]. Hattori et al. [21] indicated that these mechanical properties of Ti-Cr with 15 or 20 mass% chromium are superior to those of gold-based alloys (Type 4) and Co-Cr alloys [21]. Consequently, for clinical use as dental fixed prostheses and dental implant abutments, Ti-20Cr may possess preferable mechanical properties and a good corrosion resistance against fluoride- or peroxide- solutions [10,15,21]. Because of these advantages, the binary Ti-20Cr was investigated in this study.

Hattori et al. [21] explained that the difference of the mechanical properties compared to previous studies in Ti-Cr might lie in the cast process. The authors used an alumina/magnesia-based mold, which is similar to our procedure, because a phosphate-bonded mold induces a reaction in the titanium alloys that results in highly brittle specimens. Tschernitschek et al. [28] recommended the application of low-reactivity investment materials which contain refractory oxides (magnesium oxide, zirconium oxide, aluminum oxide, and calcium oxide) due to forming thin oxygen-enriched layers (20–25 μm). From this point of view, the authors also stated that cast processing methods and laboratory skills need to be carefully selected in order to ensure success in clinical use [28]. For biomedical applications such as orthopedic implants, a suitable balance between strength and stiffness to best match that of bone is highly essential [29]. At present, we do not evaluate the mechanical properties of Ti-20Cr. Thus, further study is needed to clarify such properties including the elastic modulus to achieve good fixation of implantation materials to the bone tissue.

Fretting corrosion is defined as a degradation process resulting from the combined action of small movements between contacting parts and the corrosivity of the environment [19]. Mechanical wear accelerates corrosion and mechanical removal from the sliding contact under tribocorrosion conditions [30]. Electrical currents are generated by corrosion behavior, resulting in electron transfer from ions in solution to the metallic surface where the reactions are occurring [24]. For Ti-Cr, with the exposure of titanium or chromium by dissolution from the oxide film, these metals are rapidly repassivated by contact with H_2_O [15]. In our study, the average activation peaks during 10 scratching cycles tended to increase in CP-Ti compared to those in Ti-20Cr. There were statistical differences between the average activation peaks for CP-Ti and Ti-20Cr at the 10 mm/s or 40 mm/s scratching speeds (*p* < 0.01). These differences may be explained by the mechanical properties of CP-Ti and Ti-20Cr, as described above [21]. The excellent mechanical properties of Ti-20Cr contributed to the resistance against mechanical surface damage, as demonstrated in this study. It has been reported that the electrochemical corrosion test using saline solution at 0.3 V showed a similar corrosion behavior for CP-Ti and Ti-20Cr, although Ti-20Cr showed higher resistance against NaF solution compared to CP-Ti [12]. Our electrochemical corrosion test against saline solution at 0.3 V was appropriate for studying the tribocorrosion behavior because the approximate potential range in the oral cavity is between −0.3 V and 0.3 V [31]. Our results clarified that the mechanical wear damage influenced and accelerated the corrosion behavior in both materials, but was more severe in CP-Ti. Therefore, the maximum activation peaks in CP-Ti (174, 229 and 343 μA) were higher compared to those in Ti-20Cr (125, 214 and 300 μA) at 10, 20 and 40 mm/s scratching speeds, respectively (Figure 3).

Interestingly, different kinetic behaviors to reach the maximum activation peak were observed between the materials during the scratching damage application. While the activation peaks of CP-Ti gradually increased and showed maximum values at the end of the second half-phase, those of Ti-20Cr showed maximum values between the first and second half-phases. One possible explanation for this phenomenon might be the influence of not only the mechanical properties but also the component of oxide passive film. In particular, the thickness of oxide films might be associated with the current intensity (or activation peak). According to a previous report using Auger electron spectroscopy (AES) [15], the thicknesses of oxide film in as-polished CP-Ti and titanium alloys containing 5 mass% chromium (Ti-5Cr) before immersion in saline solution were approximately 30 and 15 nm, respectively. Both CP-Ti and Ti-5Cr, after immersion in NaF solution, showed a thicker oxide layer than as-polished alloys owing to repassivation; however, the higher chromium content (10 mass%) in the Ti-Cr alloys yielded a thinner oxide layer on the alloy even after immersion. In addition, the authors stated that an increase in the ratio of the chromium oxide film on Ti-Cr alloys would decrease the potential for contact between the titanium oxide and fluoride in the solution. Even if a different solution was used in our study, the higher average activation peak in CP-Ti compared to that in Ti-20Cr might be due to the high dissolution of titanium from the thicker oxide films and metal debris in CP-Ti after scratching. For characterization of the repassivation kinetics in titanium, AES and X-ray photoelectron spectroscopy are valid options [32]. Unfortunately, we did not measure and clarify the concentration of dissolved metals in this study. Thus, further study is needed to investigate the dissolution of metal elements after the fretting corrosion test.

On the other hand, Komotori et al. [20] reported different behaviors of current density between the first and second half-phases after the fretting corrosion test in Ti-6Al-4V. Their results showed that current densities at low scratching speeds (below 1.0 mm/s) were similar between the first and second half-phases whereas high scratching speeds (10 and 20 mm/s) had higher current densities in the first half-phase. These conflicting results are determined by the wear mechanism at each scratching speed, resulting in the transition of wear type from abrasive to adhesive with the increase of the scratching speed. Our results disagreed with those obtained in their study because our fretting corrosion test used different titanium-based alloys, shorter scratching times (approximately 5–12 s) and fewer scratching cycles.

Faster scratching speeds showed significantly higher average activation peaks in both materials in this study (*p* < 0.05). Our scratching speeds (10–40 mm/s) were chosen based on the stride frequency of human walking, which corresponds to a scratching speed of 20 mm/s, to simulate clinical use of these materials as orthopedic implants such as artificial joints [20]. Our results disagreed with those of a previous study as described above [20]. In principle, it can be assumed that a combined process appears during scratching, consisting of an activation of the surface with an increase in the corrosion current, and on the other hand the destroyed oxide layer is simultaneously regenerated behind the contact point. Thus, the measured total corrosion current consists of the sum of the two superimposed processes. Assuming constant repassivation kinetics for a given material and environment, it follows that at a higher activation rate (scratching speed), the corrosion current will be higher. In terms of the relationship between the cumulative electric charge and duration of scratching damage application, the rate of increase in the electric charge becomes higher with the increasing scratching speed. The authors [20] stated that once destroyed, the oxide film cannot be easily regenerated at high scratching speeds. However, we disagreed with their statement because our scratching duration and repassivation times (80–378 ms) were too short.

Furthermore, the results of repassivation times at each scratching speed showed slight differences between the materials (Table 1). This was likely caused by the fact that the surface area of each specimen in this study was the same; even the average and maximum activation peaks were different within the same scratching speed. In addition, our measurements were for short periods compared to other studies. While other studies used long fixed scratching durations (2000–3600 s) [20,33], our study used fixed scratching cycles of 10 instances. Therefore, the repassivation times obtained in our study represented a quick response.

Finally, the results for the surface morphology were similar within the materials (Table 2, Figure 6). One possible explanation is that a slower scratching speed needed more scratching time. Thus, from the calculations of the total amount of the activation peak as obtained by multiplying the scratching times (Figure 5) with the respective average of the activation peak (Figure 4c), there was no difference in the materials, irrespective of the scratching speed. The total amounts of the activation peak in CP-Ti were higher than those of Ti-20Cr. Our assumption was supported by the profile depth values obtained from topography and microscopy observations (Figure 6 and Figure 7). To summarize, our results indicated that the mechanical wear damage influenced and accelerated the corrosion behavior in both materials, but it was more severe for CP-Ti.

## 5. Conclusions

Within the limitations of this in vitro study, the following conclusions were obtained:

1. The maximum activation peaks in Ti-20Cr were lower compared to those in CP-Ti in the series of scratching speed; however, different kinetic behaviors were observed between the materials.
2. The average activation peaks in Ti-20Cr were significantly lower compared to those in CP-Ti (*p* < 0.01), except at the 20 mm/s scratching speed.
3. The scratching speed also influenced the repassivation time and a faster speed showed a shorter repassivation time.
4. The profile depth of the scratched area as determined by topography observations was significantly lower in Ti-20Cr compared to CP-Ti (*p* < 0.05).

Hence, adding chromium to titanium resulted in less surface damage against simultaneous mechanical wear and electrochemical corrosion and improved the fretting corrosion resistance. 

## Figures and Tables

**Table 1:** Mean (standard deviation) of repassivation times (T_re_; ms) from the last scratching cycle for both commercially pure titanium (CP-Ti) and titanium alloys containing 20 mass% chromium (Ti-20Cr) at different scratching speeds.

Specimens	Scratching Speed		
	10 mm/s	20 mm/s	40 mm/s
Commercially pure titanium (CP-Ti)	312 (31) a,A	152 (23) b,A	108 (26) b,A
Titanium alloys containing 20 mass% chromium (Ti-20Cr)	378 (19) a,B	173 (33) b,A	80 (13) c,A

Results of statistical analysis are represented by lower and upper case letters. Different lowercase letters in the same column indicate that the groups are significantly different (*p* < 0.05). Different upper case letters in the same row indicate that the groups are significantly different (*p* < 0.05).

**Table 2:** Mean (standard deviation) of the profile depth (Pt; μm) in the scratched area of both commercially pure titanium (CP-Ti) and titanium alloys containing 20 mass% chromium (Ti-20Cr) at different scratching speeds.

Specimens	Scratching Speed		
	10 mm/s	20 mm/s	40 mm/s
Commercially pure titanium (CP-Ti)	9.20 (1.78) a,A	7.56 (0.27) a,A	7.33 (1.43) a,A
Titanium alloys containing 20 mass% chromium (Ti-20Cr)	2.01 (0.30) a,B	4.10 (1.51) a,B	3.40 (0.46) a,B

Results of statistical analysis are represented by lower and upper case letters. Different lowercase letters in the same column indicate that the groups are significantly different (*p* < 0.05). Different upper case letters in the same row indicate that the groups are significantly different (*p* < 0.05).

## References

[1] Abraham C.M. (2014). A brief historical perspective on dental implants, their surface coatings and treatments. Open Dent. J..

[2] Barão V.A., Mathew M.T., Assunção W.G., Yuan J.C., Wimmer M.A., Sukotjo C. (2012). Stability of cp-Ti and Ti-6Al-4V alloy for dental implants as a function of saliva pH—An electrochemical study. Clin. Oral Implants Res..

[3] Ohkubo C., Hanatani S., Hosoi T. (2008). Present status of titanium removable dentures—A review of the literature. J. Oral Rehabil..

[4] Kang E.H., Park S.B., Kim H.I., Kwon Y.H. (2008). Corrosion-related changes on Ti-based orthodontic brackets in acetic NaF solutions: Surface morphology, microhardness, and element release. Dent. Mater. J..

[5] Ananth H., Kundapur V., Mohammed H.S., Anand M., Amarnath G.S., Mankar S.A. (2015). Review on Biomaterials in Dental Implantology. Int. J. Biomed. Sci..

[6] Carlsson G.E., Omar R. (2010). The future of complete dentures in oral rehabilitation. A critical review. J. Oral Rehabil..

[7] Papi P., Giardino R., Sassano P., Amodeo G., Pompa G., Cascone P. (2015). Oral health related quality of life in cleft lip and palate patients rehabilitated with conventional prostheses or dental implants. J. Int. Soc. Prev. Community Dent..

[8] Sutton A.J., Rogers P.M. (2001). Discoloration of a titanium alloy removable partial denture: A clinical report. J. Prosthodont..

[9] Swaminathan V., Gilbert J.L. (2013). Potential and frequency effects on fretting corrosion of Ti6Al4V and CoCrMo surfaces. J. Biomed. Mater. Res. A.

[10] Noguchi T., Takemoto S., Hattori M., Yoshinari M., Kawada E., Oda Y. (2008). Discoloration and dissolution of titanium and titanium alloys with immersion in peroxide- or fluoride-containing solutions. Dent. Mater. J..

[11] Liu C., Zhang E. (2015). Biocorrosion properties of antibacterial Ti-10Cu sintered alloy in several simulated biological solutions. J. Mater. Sci. Mater. Med..

[12] Takemoto S., Hattori M., Yoshinari M., Kawada E., Asami K., Oda Y. (2004). Corrosion behavior and surface characterization of Ti-20Cr alloy in a solution containing fluoride. Dent. Mater. J..

[13] Zhang E., Li F., Wang H., Liu J., Wang C., Li M., Yang K. (2013). A new antibacterial titanium-copper sintered alloy: Preparation and antibacterial property. Mater. Sci. Eng. C Mater. Biol. Appl..

[14] Hsu H.-C., Wu S.-C., Wang C.-F., Ho W.-F. (2009). Electrochemical behavior of Ti–Cr alloys in artificial saliva. J. Alloys Compd..

[15] Takemoto S., Hattori M., Yoshinari M., Kawada E., Asami K., Oda Y. (2009). Corrosion mechanism of Ti-Cr alloys in solution containing fluoride. Dent. Mater..

[16] Gittens R.A., Olivares-Navarrete R., Tannebaum R., Boyan B.D., Schwartz Z. (2011). Electrical implications of corrosion for osseointegration of titanium implants. J. Dent. Res..

[17] Bhola R., Bhola S.M., Mishra B., Olsen D.L. (2011). Corrosion in titanium dental implants/prostheses—A review. Trends Biomater. Artif. Organs.

[18] Mathew M.T., Abbey S., Hallab N.J., Hall D.J., Sukotjo C., Wimmer M.A. (2012). Influence of pH on the tribocorrosion behavior of CpTi in the oral environment: Synergistic interactions of wear and corrosion. J. Biomed. Mater. Res. B Appl. Biomater..

[19] Barril S., Mischler S., Landolt D. (2005). Electrochemical effects on the fretting corrosion behaviour of Ti6Al4V in 0.9% sodium chloride solution. Wear.

[20] Komotori J., Hisamori N., Ohmori Y. (2007). The corrosion wear mechanisms of Ti6Al-4V alloy for different scratching rates. Wear.

[21] Hattori M., Takemoto S., Yoshinari M., Kawada E., Oda Y. (2010). Effect of chromium content on mechanical properties of casting Ti-Cr alloys. Dent. Mater. J..

[22] Koike M., Cai Z., Oda Y., Hattori M., Fujii H., Okabe T. (2005). Corrosion behavior of cast Ti-6Al-4V alloyed with Cu. J. Biomed. Mater. Res. B Appl. Biomater..

[23] Engelen L., Fontijn-Tekamp A., van der Bilt A. (2005). The influence of product and oral characteristics on swallowing. Arch. Oral Biol..

[24] Chen J. (2009). Food oral processing—A review. Food Hydrocoll..

[25] Goldberg J.R., Gilbert J.L. (1997). Electrochemical response of CoCrMo to high-speed fracture of its metal oxide using an electrochemical scratch test method. Biomed. Mater. Res..

[26] Upadhyay D., Panchal M.A., Dubey R.S., Srivastava V.K. (2006). Corrosion of alloys used in dentistry: A review. Mater. Sci. Eng. A.

[27] Özcan M., Hämmerle C. (2012). Titanium as a reconstruction and implant materials in dentistry: Advantages and pitfalls. Materials.

[28] Tschernitschek H., Borchers L., Geurtsen W. (2005). Nonalloyed titanium as a bioinert metal—A review. Quintessence Int..

[29] Li Y., Yang C., Zhao H., Qu S., Li X., Li Y. (2014). New developments of Ti-based alloys for biomedical application. Materials.

[30] Mischler S., Spiegel A., Stemp M., Landolt D. (2001). Influence of passivity on the tribocorrosion of carbon steel in aqueous solutons. Wear.

[31] Corso P.P., German R.M., Simmons H.D. (1985). Corrosion evaluation of gold-based dental alloys. J. Dent. Res..

[32] Hanawa T., Asami K., Asaoka K. (1998). Repassivation of titanium and surface oxide film regenerated in simulated bioliquid. J. Biomed. Mater. Res..

[33] Mischler S. (2008). Tribochemical techniques and interpretation methods in tribocorrosion: A comparative evaluation. Tribol. Int..

